# Drug‐induced liver injury as assessed by the updated Roussel Uclaf Causality Assessment Method following mild COVID‐19 in a patient under anastrozole therapy—A case report

**DOI:** 10.1002/cnr2.2028

**Published:** 2024-04-05

**Authors:** Wolfgang Lischka, Gernot Kriegshäuser

**Affiliations:** ^1^ Private Practice Gumpoldskirchen Austria; ^2^ Department of Medical Genetics Yerevan State Medical University Yerevan Armenia

**Keywords:** anastrozole, cholestatic, COVID‐19, drug‐induced liver injury, hepatocellular, updated RUCAM

## Abstract

**Background:**

Anastrozole is a selective aromatase inhibitor used for the treatment of postmenopausal hormone‐sensitive breast cancer. The major side effects include osteoporosis, hypercholesterolemia, and musculoskeletal events, such as arthralgia and myalgia. Other adverse events are rare, including symptoms of acne, masculinization, and drug‐induced liver injury, with the latter reported in a few cases only.

**Case:**

Here, we report on a patient under anastrozole therapy who developed drug‐induced liver injury as assessed by the updated Roussel Uclaf Causality Assessment Method 5 weeks after a mild SARS‐CoV‐2 infection, which is, to the best of our knowledge, the first report of its kind involving anastrozole. Discontinuation of anastrozole resulted in a marked improvement of the alanine aminotransaminase, and aspartate aminotransaminase as well as normalized lactate dehydrogenase serum levels already seen after 26 days. Surprisingly, however, the cholestatic serum markers gamma‐glutamyl transpeptidase and alkaline phosphatase showed a further rise, and took another 4 weeks to drop significantly.

**Conclusion:**

The presentation of this case is meant to alert physicians to a potential drug‐induced liver injury following mild SARS‐CoV‐2 infection in patients under anastrozole medication.

## 1 INTRODUCTION

Anastrozole is a selective aromatase inhibitor used for the treatment of postmenopausal hormone‐sensitive breast cancer.^1^ The major side effects include osteoporosis, hypercholesterolemia, and musculoskeletal events, such as arthralgia and myalgia.^2^ Other adverse events are rare, including symptoms of acne, masculinization, and drug‐induced liver injury (DILI), with the latter reported in a few cases only.^3^, ^4^, ^5^, ^6^

A significant proportion (i.e., 16%–55%) of Coronavirus Disease 2019 (COVID‐19) patients presenting with elevated liver enzymes have been reported worldwide,^7^, ^8^, ^9^ with most cases not showing specific signs and symptoms.^10^ In this respect, alanine aminotransaminase (ALT), and aspartate aminotransaminase (AST) elevation is very common in COVID‐19 patients, while increased gamma‐glutamyl transpeptidase (GGT) and/or alkaline phosphatase (ALP) is a less usual finding, mainly observed in later disease stages.^11^, ^12^

So far, most studies have focused on the prevalence of liver injury and its association with clinical outcomes in patients hospitalized for COVID‐19 pneumonia, with only a few studies focusing on the natural history and consequences of liver injury in patients with mild or asymptomatic COVID‐19.^13^ Moreover, only two cases of hepatitis and acute liver injury observed in patients with mild COVID‐19, have been described in the literature so far.^13^, ^14^

The updated Roussel Uclaf Causality Assessment Method (RUCAM) is a well‐established tool in common use to quantitatively assess causality in cases of suspected DILI,^15^ however, many publications on COVID‐19 patients described DILI in temporal association with the use of drugs but failed to evaluate the causality for drugs in these patients. Therefore, this failure certainly obscures and invalidates the published clinical features as they comprise the effects of two potential toxic compounds as confounders, namely SARS‐CoV‐2 and drugs.^16^ By systematically reviewing 996 DILI cases based on the updated RUCAM as a causality assessment method, Teschke and coworkers^16^ were able to well define DILI clinical characteristics in 393 COVID‐19 patients as well as its classification as a confounding variable.

Here, we report on a patient under anastrozole therapy who developed DILI as assessed by the updated RUCAM 5 weeks after a mild SARS‐CoV‐2 infection.

## 2 CASE DESCRIPTION

A 75‐year‐old woman underwent breast‐sparing surgery for a high‐grade invasive lobular mammary carcinoma in 2006. Tumor cells were estrogen receptor (ER), progesterone receptor (PR), HER2/neu receptor positive, and p53 negative. The patient received neoadjuvant/adjuvant chemotherapy, radiation as well as Herceptin® (trastuzumab) therapy. Furthermore, continuous anastrozole‐based antihormone therapy at 1 mg per day was introduced in 2007. No other comorbidities, routine medication, or significant family history could be identified in our patient. Furthermore no changes in habits or diet have occurred and no new dietary supplements or herbal drugs have been introduced.

**FIGURE 1:**
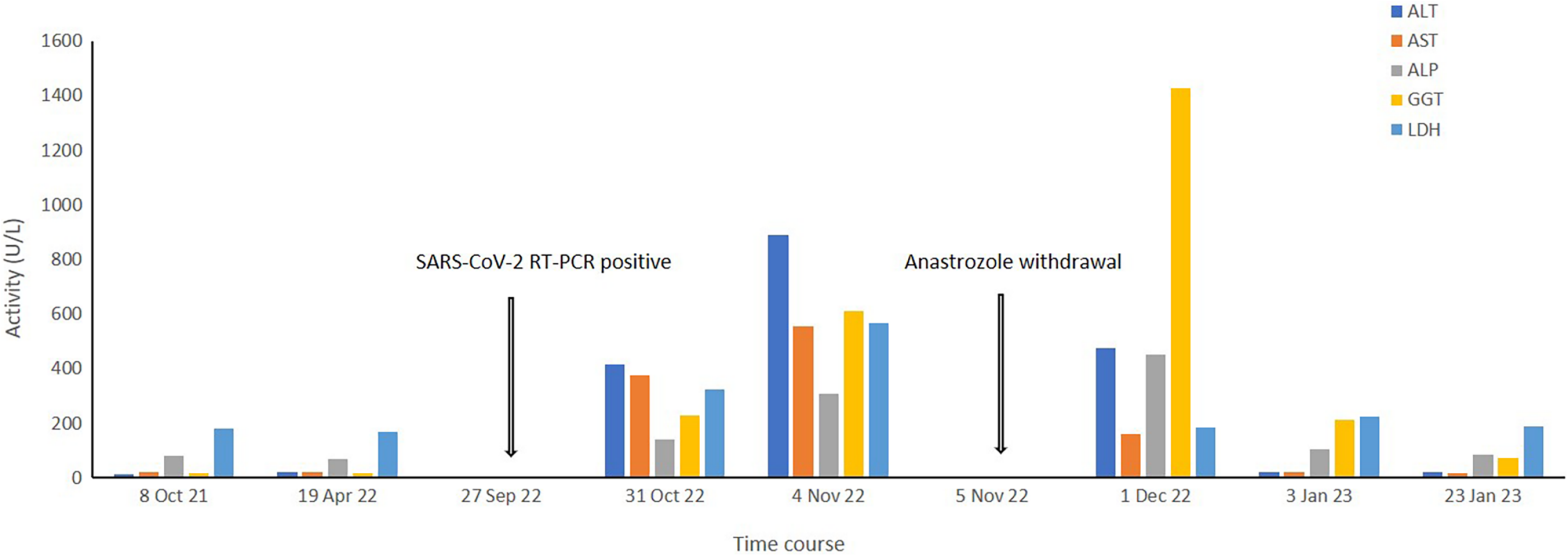
Time course of liver function tests for our patient with DILI following mild COIVD‐19.

**FIGURE 2:**
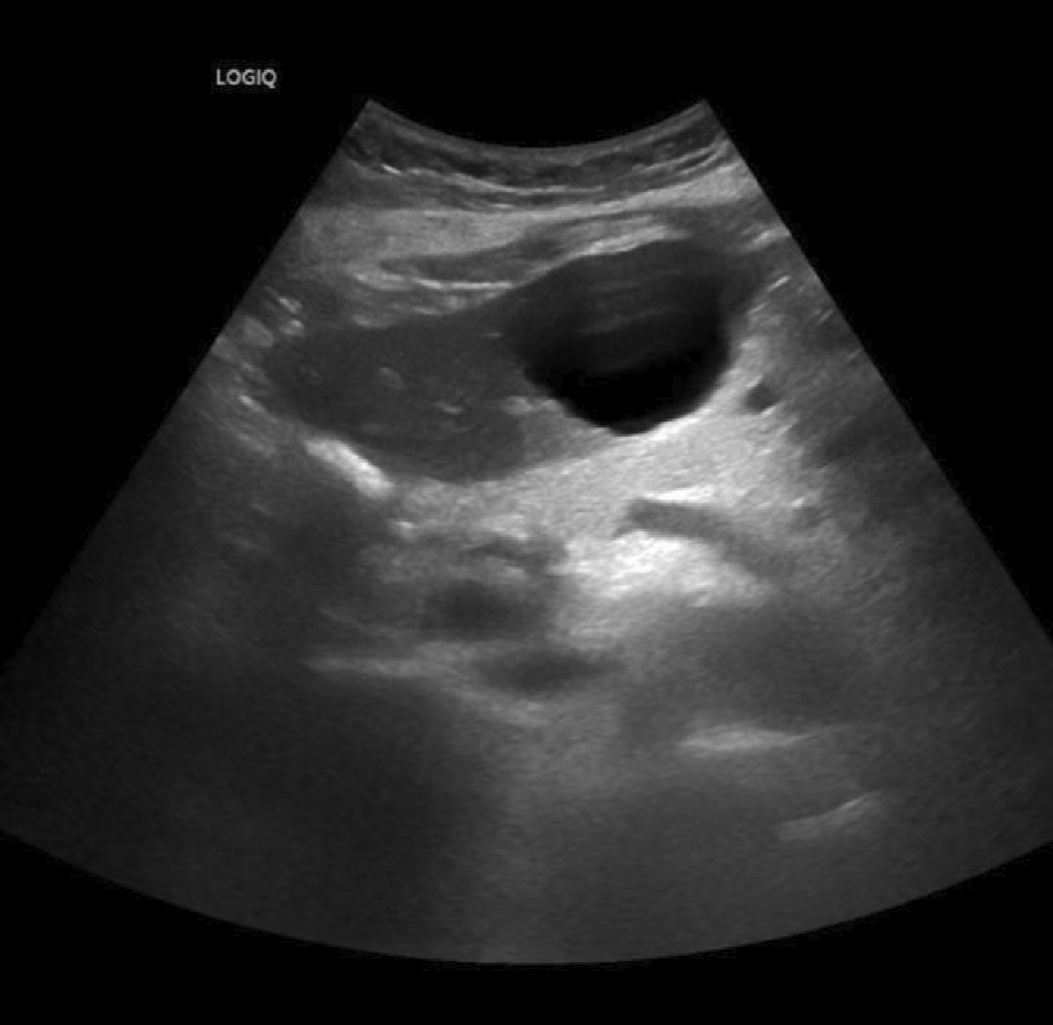
Ultrasound image showing a hypoechoic hepatic cyst (Ø 43 mm) in the left lobe.

Standard follow‐up care was established by her surgeon and over the years, her laboratory tests have always resulted in normal levels of the liver enzymes ALT, AST, ALP, and GGT. At the end of September 2022, the patient contracted a mild form of Covid‐19 disease as indicated by positive RT‐PCR nasopharyngeal swab testing for SARS‐CoV‐2. Thirty‐four days later, a routine blood test performed by her general practitioner revealed significantly elevated liver enzymes ALT 414 U/L (normal range: <35), AST 374 U/L (normal range: <35), ALP 139 U/L (normal range: <104), GGT 228 U/L (normal range: 5–40), and lactate dehydrogenase (LDH) 323 U/L (normal range: 135–214) indicating hepatocellular rather than cholestatic liver injury. With this respect, the patient denied pain, fatigue, fever, and did not suffer from jaundice. After 4 days, a follow‐up laboratory analysis indicated even higher liver enzyme levels ALT 891 U/L (normal range: <35), AST 554 U/L (normal range: <35), ALP 307 U/L (normal range: <104), GGT 611 U/L (normal range: 5–40), and LDH 567 U/L (normal range: 135–214) (Figure 1), with an updated RUCAM score of 6 indicating probable DILI. Other common causes and of liver damage (e.g., virus infection and autoimmunity) were ruled out by means of serological tests for hepatitis A, B, and C, cytomegalovirus (CMV), Epstein–Barr virus (EBV), antinuclear antibodies (ANA), antimitochondrial antibodies (AMA), smooth muscle (SM), and liver‐kidney microsomal (LKM) antibodies. Furthermore, an abdominal ultrasound yielded no pathological result with exception of a familiar liver cyst in the left lobe (Figure 2).

Because of its possible hepatotoxic effect, anastrozole was discontinued resulting in a marked improvement of AST, ALT and a normalized LDH serum level already seen after 26 days. Surprisingly, however, within a follow‐up period of 3 months, the cholestatic serum markers showed a further rise, with GGT 1427 U/L (normal range: 5–40) and ALP 451 U/L (normal range: <104) and took another 4 weeks to drop significantly (Figure 1).

## 3 DISCUSSION

Here, we report on a patient under anastrozole therapy who developed DILI as assessed by the updated RUCAM 5 weeks after a mild SARS‐CoV‐2 infection, which is, to the best of our knowledge, the first report of its kind involving anastrozole.

Anastrozole‐induced hepatotoxicity is a rare event and if it manifests, it does so within the first few months of starting this medication.^3^, ^6^, ^7^ Therefore, DILI caused by toxic or immunological mechanisms is usually not expected to appear with a latency of 15 years of medical drug consumption. No changes in our patient's habits or diet have occurred and no new dietary supplements or herbal drugs have been introduced. Liver injury is a common feature in COVID‐19 patients and is associated with disease severity and prognosis usually exhibiting mild elevation of the aminotransferases <5 times above the upper limit of normal (ULN), only.^10^ In such cases, liver injury was transient with liver function parameters returning to normal within 2–3 weeks.^11^ In contrast, increased GGT and/or ALP are less common features, mainly observed in later disease stages.^10^ Severe hepatotoxicity (i.e., aminotransferases elevation >20 times ULN) has been reported to affect 2% of hospitalized COVID‐19 patients, with acute liver failure being an extremely rare event.^17^ A recent study including 158 hospitalized patients showed, that the incidence of abnormal liver function tests (LFTs) was significantly higher in severe than in nonsevere COVID‐19 cases. Furthermore, a substantial percentage of patients still had abnormal LFTs by the time of discharge.^18^ With this respect, drugs should be considered as tentative culprits for abnormal LFTs with RUCAM‐based DILI and its clinical characteristics and classification as a confounding variable in COVID‐19 patients now being well defined.^16^ Key elements of DILI are described in condensed form: (1) DILI in the COVID‐19 study cohort was preferentially caused by antiviral drugs given empirically in face of their proven efficiency in infection diseases caused by a variety of viruses; (2) the updated RUCAM worked well even in patients with up to 18 comedicated drugs, providing clear individual causality gradings for each drug used; (3) according to RUCAM criteria using ALT and ALP as diagnostic parameters, hepatocellular injury was more often found than cholestatic or mixed injury; (4) maximum LT values were published for alanine aminotransferase (ALT) 1.541 U/L and aspartate aminotransferase (AST) 1.076 U/L; (5) the ALT/AST ratio was variable and ranged from 0.4 to 1.4; (6) the age of the COVID‐19 patients with DILI ranged from 54.3 to 56 years; (7) the ratio of males to females was 1.8–3.4:1; (8) outcome was favorable for most patients, likely due to careful selection of the drugs and quick cessation of drug treatment if DILI developed, but clinical course was related to fatal disease in one patient; (9) countries reporting RUCAM‐based DILI cases in COVID‐19 patients included China, India, Japan, Montenegro, and Spain; (10) the analyzed six reports were not designed to determine the quantitative contribution of DILI and the COVID‐19 virus in the abnormal LFTs.^16^

In our 75‐year‐old patient, we observed highly increased serum ALT and AST concentrations of >25 and >15 times ULN, respectively, with a variable ALT/AST ratio ranging from 1.1–1.6. Moreover, elevated serum ALP and GGT levels of >4 and >35 times ULN, respectively indicated the presence of mixed DILI, which is a less usual finding in patients with COVID‐19.^11^, ^12^ Moreover, only two cases of hepatitis and acute liver injury observed in patients with mild or asymptomatic COVID‐19, have been described in the literature so far.^13^, ^14^

Acute liver injury with serum ALT and AST levels >10 and >1.7 times ULN, respectively, but without cholestatic features, was reported in a patient with mild COVID‐19, and 39 days after the detection of SARS‐CoV‐2 by nasopharyngeal swab testing.^13^ However, even though this patient self‐administered Traditional Chinese Medicine (TCM), the authors did not evaluate the possibility of DILI as assessed by the updated RUCAM. In a second patient with asymptomatic COVID‐19 and not taking any medication at all, acute hepatitis with AST 1531 U/L (normal value <35), ALT 893 U/L (normal <36), serum bilirubin 1.02 mg/dL (normal <1.2), ALP 106 U/L (normal 33–98), and GGT 1276 U/L (normal <40) was reported 10 days after infection with SARS‐CoV‐2. However, AST, ALT, and GGT serum levels already dropped significantly after another 3 days.^14^

Because of the severity, the pattern of hepatotoxicity, the prolonged elevation of hepatocellular and cholestatic serum markers, and the mild COVID‐19 disease it is tempting to speculate that SARS‐CoV‐2 infection triggered DILI, a notion that is supported by an updated RUCAM score of 6 calculated for our patient. This observation is remarkable because it is, to the best of our knowledge, the first of its kind involving anastrozole. Nevertheless, it is limited by the fact that hepatitis E virus (HEV) infection was not excluded from the list of differential diagnoses as well as the lack of radiological data derived from computed tomography or magnetic resonance imaging.

In conclusion, the presentation of this case is meant to alert physicians to a potential DILI following mild SARS‐CoV‐2 infection in patients under anastrozole medication. Patients presenting with a similar condition should be identified in order to substantiate the clinical significance of our observation.

## AUTHOR CONTRIBUTIONS

**Wolfgang Lischka:** Conceptualization (supporting); data curation (lead); formal analysis (supporting); writing – original draft (supporting); writing – review and editing (equal). **Gernot Kriegshäuser:** Conceptualization (lead); data curation (supporting); formal analysis (lead); writing – original draft (lead); writing – review and editing (equal).

## CONFLICT OF INTEREST STATEMENT

The authors have stated explicitly that there are no conflicts of interest in connection with this article.

### ETHICS STATEMENT

The Center of Medical Genetics and Primary Healthcare Institutional Review Board exempted ethics approval for case reports.

### INFORMED CONSENT

Full consent for participation and publication was provided by the patient.

## Data Availability

The data that support the findings of this case report are available from the corresponsing author upon reasonable request.
